# Cerebral Cortical Thickness in Chronic Pain Due to Knee Osteoarthritis: The Effect of Pain Duration and Pain Sensitization

**DOI:** 10.1371/journal.pone.0161687

**Published:** 2016-09-22

**Authors:** Hamza M. Alshuft, Laura A. Condon, Robert A. Dineen, Dorothee P. Auer

**Affiliations:** 1 Arthritis Research UK Pain Centre, University of Nottingham, Nottingham, United Kingdom; 2 Radiological Sciences, Division of Clinical Neuroscience, University of Nottingham, Nottingham, United Kingdom; 3 Arthritis Research UK Pain Centre, Radiological Sciences, Queen's Medical Centre, Nottingham, United Kingdom; 4 Division of Rehabilitation and Aging, the University of Nottingham, Nottingham, United Kingdom; Université catholique de Louvain, BELGIUM

## Abstract

**Objective:**

This study investigates associations between cortical thickness and pain duration, and central sensitization as markers of pain progression in painful knee osteoarthritis.

**Methods:**

Whole brain cortical thickness and pressure pain thresholds were assessed in 70 participants; 40 patients with chronic painful knee osteoarthritis (age = 66.1± 8.5 years, 21 females, mean duration of pain = 8.5 years), and 30 healthy controls (age = 62.7± 7.4, 17 females).

**Results:**

Cortical thickness negatively correlated with pain duration mainly in fronto-temporal areas outside of classical pain processing areas (p<0.05, age-controlled, FDR corrected). Pain sensitivity was unrelated to cortical thickness. Patients showed lower cortical thickness in the right anterior insula (p<0.001, uncorrected) with no changes surviving multiple test correction.

**Conclusion:**

With increasing number of years of suffering from chronic arthritis pain we found increasing cortical thinning in extended cerebral cortical regions beyond recognised pain-processing areas. While the mechanisms of cortical thinning remain to be elucidated, we show that pain progression indexed by central sensitization does not play a major role.

## Introduction

Chronic musculoskeletal pain is a major health and societal burden affecting about 30% (13.5–47%) of the general population, and osteoarthritis (OA) is considered one of the main causes [1]. Over the past decade, several studies have identified brain morphometric changes in chronic pain, but the nature and causative factors of these changes are largely unknown. Grey matter (GM) losses were interpreted as either reflective of structural neuroplasticity with some evidence for reversibility, or alternatively as pre-existing trait marker of pain vulnerability [2]. Reduction in grey matter volume has been noted in several chronic pain states [3]. However, the distribution of pain-related GM changes varies greatly between studies, even when studying identical primary aetiologies of chronic pain disorders.

It remains unclear whether maladaptive or neuroplastic changes during pain progression contribute to structural brain findings in chronic pain. Some studies have reported negative correlations i.e. less GM volume with longer pain duration [4, 5], while others found positive [6, 7], or no correlation [8, 9]. These discrepancies may reflect a random error (small sample sizes), technical limitations of GM volume estimation, non-linearity of the interrelation or true biological differences. Moreover, the multidimensionality of chronic pain and related comorbidities may underlie the heterogeneity in the observed pattern of grey matter changes among different pain states. However, independent of the precise mechanism causing brain volume reduction in chronic pain, one can argue that there are two possible association patterns: Morphometric changes linked to innate pain vulnerability will be pre-existing and are unlikely to progress, while changes in response to the chronic pain experience will be cumulative and hence be associated with pain duration. The latter association pattern would be a strong indicator of maladaptive neuroplasticity underlying morphometric brain changes in chronic pain.

Central sensitization is one of the key mechanisms underlying pain chronification that might also explain structural brain plasticity associated with chronic pain. It is defined by the International Association for the Study of Pain as “an increased responsiveness of nociceptive neurons in the central nervous system to their normal or sub-threshold afferent input” [10]. In contrast to peripheral sensitization, which involves local pain hypersensitivity restricted to the damaged area, central sensitization results in remote pain hypersensitivity in non-injured areas [11], and may persist without clear peripheral nociceptive conduction. Reduced pain thresholds at body sites remote from the primary clinical pain site as a sign of central sensitization can be demonstrated in several chronic pain conditions [12–14], including osteoarthritic pain [11]. Quantitative sensory testing using a pressure algometer to detect local and remote mechanical pain thresholds is a commonly used non-invasive tool to quantify sensory and pain perception in both normal and chronic pain conditions [11, 15].

Putative links between central sensitization and brain morphometry in chronic pain are under-researched with preliminary contradictory findings. In fibromyalgia for instance, grey matter volume loss in anterior cingulate and prefrontal cortex showed moderate correlation with central sensitization [16]. In contrast, no associations were found between cortical thickness and cutaneous heat pain thresholds in migraine patients [17]. As far as we are aware there are no studies that assess central sensitization and cortical morphometric changes in primarily nociceptive pain states.

Nevertheless, there is some evidence that inter-individual differences in pain sensitivity in healthy controls are partly explained by cortical thickness in the left superior temporal/inferior parietal region with thinner cortex associated with higher pain sensitivity [17]. Brain morphometric associations with pain hypersensitivity may be modality-specific with cortical thickness of SI positively correlated with heat pain sensitivity, and thickness of the paracentral lobule with cold pain sensitivity [18]. Cortical thickness in the anterior cingulate in that study was also noted to be associated with increased sensitivity to warm detection [18]. Importantly, grey matter loss was observed in pain sensitizers after repeated pain exposure in several brain areas including the anterior cingulate cortex (ACC) [19]. Compelling evidence for a role of the ACC in central sensitization comes from several studies in preclinical models of chronic pain [20]. We thus hypothesize that central sensitization may explain cortical thinning in chronic pain predominantly affecting the ACC.

Pain in osteoarthritis is considered primarily nociceptive initially resulting from joint tissue damage, but additional central mechanisms are increasingly recognized during pain progression [21]. Predominant central pain might explain why around 15% of patients undergoing knee replacement surgery will not achieve pain relief [22]. A recent meta-analysis confirmed reduced pain thresholds in OA patients at affected and remote body sites suggesting the presence of both peripheral and central sensitization [11]. There is also evidence for impaired central pain modulation and increased temporal sensitization in chronic OA pain [23] further demonstrating central pain mechanisms.

Progress in brain morphometric methods enabled surface based approaches to more reliably quantify cortical structure and distinctly study the genetic and phenotypical underpinning of cortical thickness from surface area changes that both contribute to grey matter changes [24]. This method has gained popularity to investigate cerebral morphometry in chronic pain [2], [25]. There are several advantages of the surface-based approach over standard volumetric methods: cortical thickness assessment provides a directly interpretable metric, allows for detection of sub-voxel changes [26], while being less sensitive to inaccuracies of spatial normalization and smoothing [27], and has been well validated [28].

Here, we assess cortical thickness using surface-based measurements of high-resolution 3T brain MRI in chronic knee OA pain subjects to test the primary hypotheses (i) that cortical thinning progresses over the duration of chronic pain, and (ii) that central sensitization indexed as increased mechanical pain sensitivity remote from the affected joint is associated with cortical thinning primarily in the ACC and other pain processing areas.

## Materials and Methods

### Subjects

Patients (*n* = 40, age = 66.09± 8.47; 21 females) with radiologically defined knee OA were included if they had chronic pain defined as ‘pain lasting for ≥3 months and experienced most of the day on most days of the week for at least the last month [29], (Table 1 and S1 Table). Patients were identified via general practices, orthopaedic outpatient clinics, and from previous local studies. Exclusion criteria were major medical, neurological or psychiatric co-morbidities, under 18 years, pregnancy, previous joint replacement or MRI contraindications.

**Table 1:** Characteristics of study participants.

		Age (Y)	Sex (M: F)	BMI	Handed-ness (R: L)	Educ. level 1–8	MoCAMax. 30	BDI 0–63	Pain duration (Months)	Ch. Pain severity 0–10	VAS 0–10	MPQ Sensory 0–42	MPQ Affective0-14	Pain-DETECT 0–38
***Patients n = 40***	***Mean***	66.09		28.8		*3*.*9*	*26*.*8*	*7*.*3*	*102*.*1*	5.4	3.2	12.5	*1*.*4*	11.4
	***SD***	8.47		4.9						5.5		12		12
	***Median***			27.4		3	27	7	72		3		1	
	***Range***	45.4–83.0		20–41.1		1–8	18–30	0–22	12–456	1–10	0–9	0–31	0–11	0–25
	***Ratio***		19:21		37:2 (1NA)									
***HC n = 30***	***Mean***	62.72		26.21		*5*.*6*	*27*.*2*	*2*.*2*						
	***SD***	7.44		4.9				
	***Median***			25.3		6	27	1
	***Range***	46.8–80		18.9–41.4		1–8	23–30	0–12
	***Ratio***		13:17		27:3			
***P-Value***		**.*09***^***§***^	**.*73***^***Φ***^	**.*04***^***§***^	**.*64***^***Φ***^	**.*07***^***Φ***^	**.*9***^***¶***^	**.*001***^***¶***^

BMI = Body Mass Index, Education scores based on 8 categories represent the British education system levels where 1 = none and 8 = higher degrees [modified from [30]], MoCA = Montreal Cognitive Assessment. BDI = Beck’s Depression Inventory. Ch. Pain severity = chronic pain severity; average pain intensity over the past four weeks on a scale from 0 to10. VAS = visual analogue scale.

§ = Independent t-test.

¶ = Independent samples Mann-Whitney U Test.

Φ = Chi-squared Tests or Fisher’s Exact Test when sample size is <5 in any cell.

Pain-free healthy controls (*n* = 30, age = 62.72± 7.44; 17 females) were recruited via posters and through invitations to patients’ partners. All healthy controls were confirmed non-OA via general practitioners database. Written informed consent was obtained from all participants according to the declaration of Helsinki, and Nottingham Research Ethics Committee 2 NHS approved the study (10/H0408/115).

Pain characteristics were assessed in patients using the self-administered McGill pain (MPQ) [31] and PainDETECT [32] questionnaires that record sensory and affective, and neuropathic pain components respectively. Pressure pain thresholds (PPT) were recorded by a trained single rater using a digital algometer (Somedic AB, Sweden) with three repetitions at the index finger, sternum, medial tibia, and at the medial and lateral joint lines of the painful knee. The average of the last two readings for the three non-local sites was used to indicate non-knee or remote PPT, and those from knee joint lines to indicate knee or local PPT. Cognitive ability of all participants was evaluated during a face-to-face interview using the Montreal Cognitive Assessment test (MoCA) [33]. Depressive symptoms were assessed using Beck’s Depression Inventory BDI-II [34]. Educational level [30], handedness[35], body mass index (BMI) and medications were recorded in all participants (Table 1) and S1 Table.

### Brain Magnetic Resonance Imaging

MRI was performed at 3T (Discovery 750, GE Medical Healthcare, Milwaukee, US) using a 32-channel head coil. 3D anatomical brain scans were acquired using axial T1 FSPGR-BRAVO sequence (Voxel size = 1mm^3^, TE = 3.3ms, TR = 8.5ms, TI = 450, FA = 12, Acceleration factor = 2, acquisition time = 4min 10s).

### Cortical thickness analysis

Cortical reconstruction and thickness estimation (CT) was performed using the Freesurfer software package (Mac version 5.1.0 available at (www.surfer.nmr.mgh.harvard.edu), following standard procedures and rigorous visual quality control. Briefly the processing involves removal of non-brain tissue, transformation into Talairach space, intensity normalization, tessellation of the grey matter white matter boundary, automated topology correction, and surface deformation is then performed to indicate the grey/white and grey/cerebrospinal fluid borders based on detection of greatest shift in intensity [36]. The method uses both intensity and continuity information from the entire three dimensional MR volume in deformation procedures to produce representations of cortical thickness, calculated as the closest distance from the grey/white boundary to the grey/CSF boundary at each vertex on the tessellated surface. The reconstruction output is then visually inspected and any parcellation inaccuracies were manually corrected as per Freesurfer manual. This process involved all subjects to ensure that the automated parcellations follow the actual surface of the cortex. In all subjects some degree of manual correction was required ranging from as minimal as 2 consecutive brain slices (1 slice = 1mm) to a maximum of 16 non-consecutive slices. This was performed and checked in coronal, sagittal and transverse views to ensure accuracy by one trained researcher while blinded to the clinical details (HA) supervised by an experienced neuroradiologist (RAD). The maps produced are not restricted to the voxel resolution of the original data thus are capable of detecting changes at sub-millimetre levels.

### Statistical analysis

We used SPSS version 20 for demographic and global association statistical analysis. Independent t-test was used to examine group differences in parametric data of age, BMI, pain severity, sensory and affective components of pain, and Mann-Whitney U tests to examine group differences in scores of the non-parametric data of MoCA, BDI, pain duration and PainDETECT. Between group comparisons of sex, handedness and educational status were assessed using Chi-Squared tests.

To test our main hypotheses that cortical thinning is correlated (i) with pain duration, and (ii) with remote mechanical pain sensitivity general linear mixed models implemented in QDEC (Freesurfer statistical and visualization tool; www.surfer.nmr.mgh.harvard.edu) were used. To this end, we performed whole-brain surface based correlation (pain duration and PPT) analysis controlled for age. We did not control for intracranial volume, as cortical thickness different from cortical volumes does not scale with differences in intracranial size. The dependence of regional volumes from intracranial size largely results from surface area, however this does not apply to cortical thickness, and in fact no association could be demonstrated between head size and cortical thickness [37].

Due to the skewed distribution we used log-transformed data for pain duration where a simple logarithmic (z = log to base 10 y) transformation was made on the raw data to normalize the distribution. Significance was set at p<0.05 (corrected for multiple comparisons using false discovery rate FDR). To account for our regional hypothesis for the pain sensitivity analysis, small volume correction was used deploying a mask that included dorsal ACC using the Freesurfer parcellation atlas aparc.a2009s [38], with a significance threshold set at corrected p<0.05 using Monte Carlo permutations with 5000 iterations using AlphaSim (http://afni.nimh.nih.gov/afni/doc/manual/Alpha-Sim).

Secondary confirmatory statistical tests were performed to study associations of averaged cortical thickness and pain duration and pain sensitivity using parametric or non-parametric correlation analysis as appropriate. To confirm previously reported morphometric brain changes in OA patients we used QDEC and general linear mixed models with a between group design, corrected for age. This included a subgroup analysis of patients after applying a median split into shorter (less than 6 years, SPD) and longer pain duration (6 years or longer, LPD) in line with a previous study also using a median split suggesting that brain morphological changes may only become discernible after more than five years [39]. Results for these between group comparisons are reported at uncorrected p<0.001, but all tests were controlled for age to allow direct comparison with existing literature. Lastly, we compared averaged cortical thickness between patients and controls (using age corrected ANCOVA, implemented in SPSS) to explore putative factors that may generally predispose to low cortical thickness.

## Results

### Demographic data

Patients with knee osteoarthritis (KOA) and healthy controls (HC) did not differ in age and sex. Detailed participants’ characteristics are shown in Table 1. KOA suffered from chronic knee pain as their main complaint (21 with dominant right, and 19 with dominant left knee pain), and had not undergone knee or other joint replacement. 19 out of 40 patients were on regular medication with a very similar frequency for the short and long pain duration subgroups: anti-hypertensive drugs (n = 9 (total); 5 LPD, 4 SPD) or non-opioidergic painkillers (n = 8 total; 4 LPD: 4 SPD), S1 Table for more details. Healthy controls were pain-free and non-medicated except three subjects were on Zopiclone for insomnia, eye drops for glaucoma and oestrogens, respectively.

Compared to healthy controls, KOA patients presented with higher scores of self-reported low mood (p = 0.001, Table 1), ranging from minimal to the upper limit of mild depression [40]. There was no significant difference in the cognitive ability between patients and healthy controls with the latter showing non-significantly higher educational levels, and significantly less body mass index. There was no significant difference between short and long pain duration patient strata in age, sex distribution, cognitive ability or mood.

### Pain characteristics

Most patients described nociceptive or unclear pain symptoms according to PainDETECT scores (0–12: ‘nociceptive’, *n* = 24, 60%, 13–18 ‘indeterminate’, *n* = 11, 27.5%), with only five (12.5%) considered to have neuropathic-like symptoms (PainDETECT scores 19–38). Patients with short as compared to those with long pain duration presented with significantly higher reports of chronic pain severity and also a tendency for higher pain intensity on the day of scanning. Higher pain intensity in the SPD group was associated with a higher MPQ affective domain score but normal sensory MPQ component (Table 2).

**Table 2:** Demographic data and pain characteristics of short vs. long pain duration groups.

		Age (Y)	Sex (M: F)	Pain duration (Months)	Ch. Pain severity	Pain severity (VAS)	Sensory (MPQ)	Affective (MPQ)	Pain-DETECT	BDI
					0–10	0–10	0–42	0–14	0–38	0–63
***SPD (n = 20)***	***Mean***	***65*.*2***		***33*.*9***	***6*.*3***	***3*.*9***	***13*.*8***	***1*.*9***	***10*.*6***	***6*.*7***
	***SD***	8.2			2.4	4.2	7.7		6.8	
	***Median***			33				1		4.5
	***Range***	45.4–83		12–60	2–10	0–9	0–31	0–11	0–23	0–22
	***Ratio***		7:13							
***LPD (n = 20)***	***Mean***	***66*.*9***		***165***	***4*.*4***	***2*.*5***	***11*.*2***	***0*.*9***	***12*.*2***	***8*.*1***
	***SD***	8.8			4.5	2.4	8.1		5.6	
	***Median***			120				0		7.5
	***Range***	48.3–81		72–456	1–8	0–7	0–29	0–7	1–25	0–19
	***Ratio***		***12*:*8***							
***P-value***		***0*.*5***^***§***^	***0*.*2***^***Φ***^	***< 0*.*001***^***¶***^	***0*.*01***^***§***^	***0*.*07***^***§***^	***0*.*3***^***§***^	***0*.*04***^***¶***^	***0*.*4***^***§***^	***0*.*2***^***¶***^

SPD = Short Pain Duration, LPD = Long Pain Duration, VAS = Visual Analogue Scale, MPQ = McGill Pain Questionnaire, BDI = Beck Depression Inventory.

§ = Independent t-test.

¶ = Independent samples Mann-Whitney U Test.

Φ = Fisher’s Exact Test.

### Quantitative Sensory Testing (pressure pain)

One KOA dataset was discarded due to a technical error. Knee PPT were significantly lower in KOA vs. HC (p = 0.002, Table 3), but there was only a borderline trend (p = 0.1) for lower PPT in KOA at remote sites. Neither knee nor remote PPTs correlated with chronic pain severity or duration.

**Table 3:** Pressure pain threshold measurements for KOA vs. HC.

	Average knee PPT KPa (M± SD)	Average non-knee PPT KPa (M± SD)
**Knee OA** (*n* = 39)	308.9± 173.6	237.4± 114.2
**HC** (*n* = 30)	467.5± 232.3	283.6± 116.8
***P* value**	**0.002**	**0.1**

### Cortical thickness findings in chronic painful knee OA compared to healthy controls

The average CT did not significantly differ between KOA and HC controlling for age (2.37± 0.1 and 2.42± 0.08 respectively, p = 0.08; ANCOVA).

Whole brain vertex-based analysis did not reveal significant differences at FDR corrected level. At the uncorrected p<0.001 level controlled for age, thinner cortex was found in the right anterior insula in KOA. No differences were seen for the left hemisphere.

### Long and short pain duration

There was no difference in average cortical thickness between long and short pain duration groups, controlled for age.

Patients with long but not with short pain duration showed thinner left precuneus cortex (p<0.001 uncorrected, controlled for age) when compared to healthy controls.

### Correlations of cortical thickness with pain duration in chronic painful knee OA

Average CT of the total brain, correlated inversely with the log-transformed data of pain duration with moderate strength *r* = -0.46 (p = 0.01, controlled for age).

**Fig 1:**
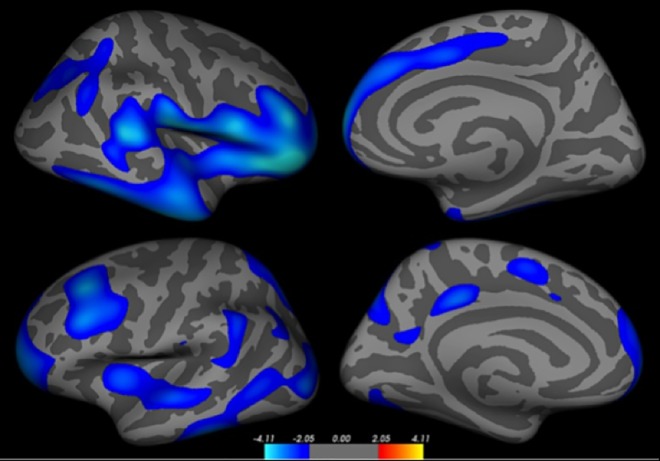
Cortical surface maps showing thinner cortex with log-transformed longer pain duration (right hemisphere; top, and left hemisphere; bottom). Significance at p<0.05 FDR-corrected for multiple comparisons and controlled for age.

Detailed vertex-based whole brain correlation analysis revealed extended bilateral areas involving several clusters (ranked according to Z-score, Fig 1 and Table 4) where log-transformed pain duration correlated negatively with cortical thickness i.e. thinner cortex with longer pain (p<0.05, FDR corrected). As some clusters spanned over several regions, sub-clusters were detailed and can be found in S2 Table. No positive correlations were seen at corrected nor at uncorrected p<0.001 levels.

**Table 4:** Clusters of significant negative correlation with pain duration reported at the peak coordinates, (Rt. & Lt. hemispheres).

Cluster*	Max	Tal X	Tal Y	Tal Z	Brodmann Area	Anatomy
R 1	-4.76	14.7	76.9	-30.2	47	Pars orbitalis
2	-2.38	31.3	-63.4	36.8	19	Inferior parietal
L 1	-3.59	-29.7	24.8	37.8	9	Rostral middle frontal
2	-3.28	-21.6	57.1	-13.2	11	Rostral middle frontal
3	-3.28	-50.3	-40.7	-25.0	21	Inferior temporal
4	-3.03	-5.5	-32.1	39.9	31	Posterior cingulate
5	-2.86	-16.7	-76.0	44.2	7	Superior parietal
6	-2.82	-38.1	-2.6	1.1	-	Insula
7	-2.56	-54.1	-47.3	37.8	40	Supramarginal
8	-2.53	-8.4	0.1	53.2	6	Superior frontal
9	-2.14	-32.7	-68.2	43.4	7	Inferior parietal
10	-2.08	-6.4	-61.5	17.2	30	Precuneus
11	-1.91	-11.1	13.5	34.5	24	Caudal anterior cingulate
12	-1.88	-57.3	-22.8	22.6	40	Supramarginal

*At peak co-ordinate

### Post-hoc region-of-interest CT analysis

**Fig 2:**
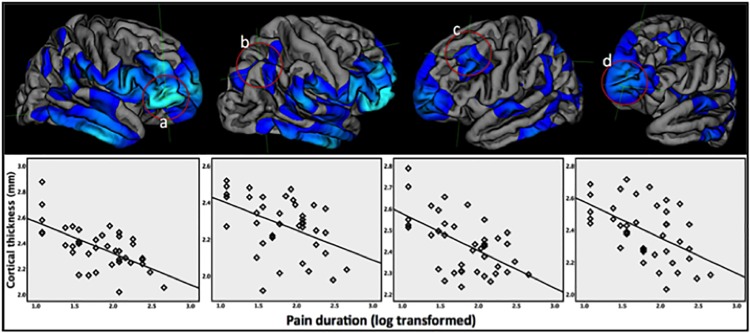
Scatter plots showing negative correlations of cortical thickness with log-transformed pain duration for four regions showing strongest associations (a-d; right pars orbitalis and inferior parietal, and left rostral middle frontal and frontal pole, respectively).

To illustrate the association of cortical thinning with the duration of chronic pain, we plotted peak coordinates of four regions that showed strongest association between CT and pain duration (Fig 2) highlighting linear correlation with log transformed pain duration, (correlation with pain duration raw data is provided in S1 Fig). Using partial correlations we further explored whether mood scores or pain sensitivity moderated the interrelations. We found that the increasing cortical thinning for these ROIs over the duration of pain remained independently significant after controlling for the partial effects of mood and pain sensitivity scores at p<0.003.

### Central sensitization and anterior cingulate cortical thickness

Thickness of the anterior cingulate cortex was not associated with central sensitization (partial correlation: r = 0.25, p = 0.19, corrected for multiple comparisons based on Monte Carlo permutations with 5000 iterations).

We also did not find any significant correlation between remote pressure pain thresholds and CT at vertex-level or globally (p<0.05 FDR, age corrected).

Our patients showed only non-significant lower pressure pain thresholds remotely, but significantly reduced pain thresholds at the affected knee, which indexes predominant peripheral sensitization. We thus, repeated the analysis using local pressure thresholds, which again did not demonstrate any association with cortical thickness in the ACC or globally.

## Discussion

We found global and widespread regional cortical thinning over the duration of several years of chronic pain in knee OA pain patients. Affected areas extended beyond classical pain processing areas, and findings were not explained by central sensitization or low mood. Only the right anterior insular cortex was thinner in OA patients relative to controls, but the effect did not survive multiple test correction.

Longer pain duration in knee OA patients was associated with extended, bilateral cortical thinning after controlling for age effects using whole vertex-wise brain analysis. This link between cerebral morphometric changes and chronicity of pain symptoms strongly supports our hypothesis that morphometric changes are not predisposing factors but acquired as a result of the chronic pain experience. Apkarian and co-workers were the first to propose the notion that chronic pain induces neocortical atrophy [4]. Interestingly, while several papers report on grey matter density or volume loss assumed to accumulate with pain chronification, the association with pain duration has remained controversial. No previous study in OA established significant correlation between pain duration and grey matter density controlling for age, which may be due to smaller range of, and generally shorter duration of pain in previous study cohorts. A subgroup analysis [39] suggested that grey matter changes in multiple brain areas may only emerge after several years of persistent pain which is well in line with our subgroup analysis that patients with pain duration shorter of 6 years did not show CT abnormalities compared to healthy controls even at the liberal p<0.001, uncorrected threshold. Moreover, the observed strong interrelation of brain morphometric changes with pain duration in our data may be due to the wide range of pain duration extending to many years in our sample and possible enhanced by non-linear effects as we used log-transformed data on pain duration. Increased sensitivity and robustness of the cortical thickness analysis used in this study may be another explanation for the lack of previous studies to demonstrate this effect. Also, our findings are generally well in line with studies reporting negative correlations between pain duration and GM volume, density or cortical thickness in other chronic musculoskeletal pain disorders, such as back pain [4, 5]; globally and regionally within thalamus and dorsolateral prefrontal (DLPF), rheumatoid arthritis [2]; within thalamus, and in fibromyalgia [41]; globally and [42]; within the middle temporal and anterior cingulate gyri.

The functions associated with structures that showed most significant thinning with longer pain in the studied patients are of particular interest. The frontal pole was reported to respond to tonic noxious stimulation [43], but is also involved in joint attention [44]. Studies suggest that the rostral middle frontal gyrus is also involved in decision-making [45]. The right pars orbitals is associated with decision making involving reward, behavioural and motor inhibition [46].

The right inferior parietal cortex extending largely over Brodmann area 19 is associated primarily with visual processing [47], however surrounding areas (Brodmann 5&7) that were also affected by thinning vs. duration of pain, have been related to pain perception and were found to show activation during sensory and motor tasks including thermal pain as well as with vibrotaction and motor performance [48].

The cluster spanning over left Brodmann area 9 largely corresponds to the left dorsolateral prefrontal cortex. There is a good evidence to suggest that the dorsolateral prefrontal cortex participates in a neural network involved in pain processing with a conceivable modulatory role in nociceptive transmission [37]. Repetitive trans-cranial magnetic stimulation of left prefrontal cortex in healthy subjects showed a significant increase in thermal pain thresholds [49], supporting its proposed inhibitory function during pain perception. The findings of thinning in the dorsolateral prefrontal cortex are compatible with a key finding of reduced grey matter density in the bilateral dorsolateral prefrontal cortex in chronic back pain patients [4], and affected left DLPFC in another surface-based morphometric study of low back pain patients [25], and notably in chronic pain due to hip OA [50]. The nature of such grey matter or thickness reduction in dorsolateral prefrontal cortex and their functional consequences remains unclear, especially whether such a change would lead to functional disturbance of the inhibitory role of the DLPFC as a putative contribution to pain persistency. Interestingly, a case-control study [51] of proton magnetic resonance spectroscopy revealed reductions of N-acetyl aspartate (NAA) (relative to creatine/phosphocreatine complex) in the left dorsolateral prefrontal cortex in patients with chronic back pain, but not in the cingulate, insula, thalamus or sensorimotor regions. NAA is known to be localized primarily within neurons and is widely used as a marker for functional and structural neuronal integrity. Its relative reduction in the dorsolateral prefrontal cortex in chronic pain patients [51] may index atrophy or reversible neuropil reduction both compatible with the cross-sectional morphometric studies. Longitudinal studies showed reversibility of chronic-pain related reductions in the dorsolateral prefrontal cortex following therapeutic pain relief in chronic back pain [25], and in chronic hip OA pain [50]; thus further supporting reversible maladaptive reduction of neuropil in DLPC.

The observed pattern of cortical thinning shows only partial overlap with the pain processing areas and regions for which reversibility of morphometric findings were found after pain relief [25, 50, 52]. While at first counter-intuitive, a spatial dissociation may be explained by the multidimensionality of chronic pain experience and related comorbidities. Chronic OA pain can result in reduced quality of life [53], reduced exercise, impaired sleep and long-term medication that may contribute to the pattern of progressive cortical thinning. In our studied patients, medication intake was limited to mostly antihypertensive or painkillers in the form of regular paracetamol tablets. Of note, none of the patients were on regular opioids which reportedly can affect grey matter [54], however we are not aware of any published data that would associate paracetamol with structural brain changes. Moreover, the subgroups of patients with long and short pain duration differing in morphometric changes reported a similar frequency of antihypertensive and painkiller medication intake.

We noticed that cortical thinning with increasing duration of chronic OA pain in the studied cohort overlapped partially with the default mode network (DMN). The DMN is increasingly recognized as a core brain network related to homeostasis and introspection as opposed to task-oriented brain functions. The DMN shows reduced brain activity during experimental pain conditions as during most other tasks [55]. Moreover, during rest DMN functional connectivity appears to be disrupted in a number of chronic pain conditions including musculoskeletal [56]. This is thought to reflect an abnormal state of pain directed self-referential thought [3]. Taken together our novel findings of cortical thinning with increased chronic pain duration in parts of the DMN provide further evidence for detrimental effects of chronic pain beyond nociceptive systems and might explain the functional disruption in long distance neural networks.

Quantitative sensory testing has been widely used to characterize sensory and pain perception in both normal and chronic pain conditions [11], yet little is known about its neuroanatomical especially cortical correlates in painful states. In our studied patients we did not find any significant association between higher pain sensitivity at remote sites indexing central sensitization and global or anterior cingulate cortical changes. This is in line with negative correlative findings between heat pain thresholds and grey matter volumes in migraine [17]. However, it is important to note that the group levels of mechanical pain thresholds at remote sites in our KOA cohort were only non-significantly lower (p = 0.1) than those recorded from the healthy control group. Hence, we can infer that the observed cumulative widespread cortical changes in our cohort are not driven by central sensitization. Our results also do not support our hypothesis that central sensitization can explain ACC thinning, but due to the lack of unequivocal central sensitization of our patient cohort we cannot exclude the possibility that such correlations may exist in chronic pain patients with established central sensitization. In contrast, our patients showed clear signs of peripheral sensitization but we were unable to find any association between local pressure pain thresholds and cortical thickness either.

This study revealed only minor focal changes in cortical thickness in knee OA patients compared to healthy controls that failed to maintain significance after controlling for age and multiple testing. At uncorrected p<0.001 we found reduced cortical thickness in the right anterior insula. This is however one of the brain areas most commonly reported to show less grey matter (at the uncorrected level of p<0.001) in several chronic pain conditions including knee OA and chronic regional pain syndrome [39], hip OA pain [25] and [50], fibromyalgia [41], and myofascial temporo-mandibular pain [6] compared to healthy controls. Anterior insular cortex is further recognized for its role in a wide range of conditions including negative affect and depression [57]. Altered activity within the anterior insular cortex has also been detected in depressed patients [58], hence the observed anterior insular cortical thinning may in part be related to the negative affect associated with chronic pain. It is noteworthy that the reported right anterior insular cortical thinning in our studied cohort was part of a cluster that correlated inversely with pain duration. This points towards an acquired rather than predisposing nature of the insular morphometric changes, and it is tempting to speculate that they may result from increasing distress and aversiveness experienced over several years of chronic pain.

### Limitations

Although our study is one of the largest single centre studies of cortical thickness in chronic pain, we however, cannot exclude a type 2 error based on the limited number of subjects studied. The sample size of our study was powered to detect 0.2 mm cortical thickness changes [59], and compares favourably with most previous morphometric studies, but might have precluded the detection of subtle morphometric abnormalities compared to controls. Hence, in line with previous publications on brain morphometry in pain, we also report the between group comparison at the uncorrected p<0.001 level. Nevertheless, cortical thickness analysis as presented here is considered advantageous over previously used voxel based morphometry (VBM) studies, which we confirmed by repeat VBM analysis revealing no significant within or between group findings (results not shown), but a trend for GM reductions with longer pain duration in the posterior parts of the brain overlapping with the significant CT results.

As any cross-sectional study, the identified associations do not allow making inferences on causal relationships, and should be confirmed by longitudinal studies.

## Conclusion

In conclusion, in this cross-sectional study patients with knee OA pain showed increasing neocortical thinning in areas extending beyond classical pain processing areas with longer duration of chronic pain independent from age, and pain sensitivity. This points to neuroplasticity changes unrelated to known mechanisms of up-regulated nociception.

Despite a group size of 70 subjects and advanced morphometric technique, we could not confirm previous claims of major grey matter losses in pain-processing areas in OA and musculoskeletal pain. We only found right anterior insula cortical thinning (at uncorrected p<0.001) consistent with previous reports thus suggesting a possible role of the right anterior insula in chronic OA pain in line with its putative general role in ‘distress’ conditions.

## Supporting Information

S1 Fig Scatter plots showing negative correlation with pain duration in years for four regions (a-d; right pars orbitalis and inferior parietal, and left rostral middle frontal and frontal pole, respectively) used for region of interest analysis defined from peak coordinates for clusters with strongest negative correlation with log-transformed pain duration.
(DOCX)

S1 Table Characteristics of Knee OA patients. (DOCX)

S2 Table Detailed description of regions that exhibited significant negative correlation with pain duration in knee OA patients. (DOCX)
