# Supplementary material for: Cerebral Cortical Thickness in Chronic Pain Due to Knee Osteoarthritis: The Effect of Pain Duration and Pain Sensitization
Source: PLoS One. 2016 Sep 22;11(9):e0161687. doi: 10.1371/journal.pone.0161687 (PMC5033394; doi:10.1371/journal.pone.0161687)
Supplement: S1 Fig — Scatter plots showing negative correlation with pain duration in years for four regions (a-d; right pars orbitalis and inferior parietal, and left rostral middle frontal and frontal pole, respectively) used for region of interest analysis defined from peak coordinates for clusters with strongest negative correlation with log-transformed pain duration. (DOCX) [file pone.0161687.s001.docx]

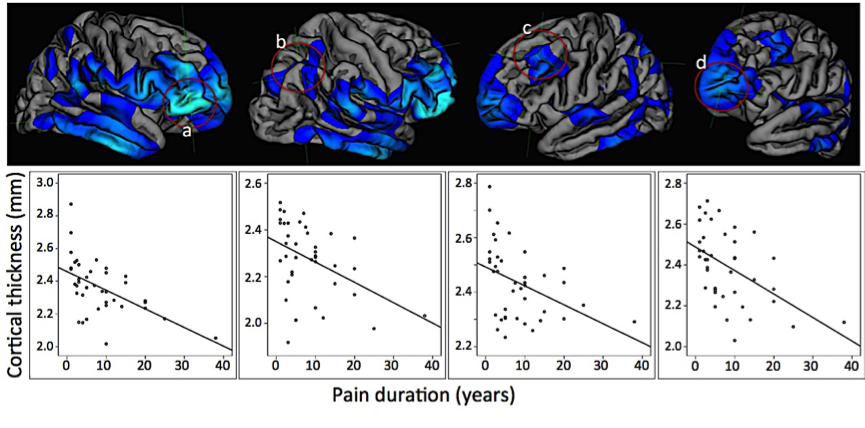


Supplementary Figure 1: Scatter plots showing negative correlation with pain duration in years for four regions (a-d; right pars orbitalis and inferior parietal, and left rostral middle frontal and frontal pole, respectively) used for region of interest analysis defined from peak coordinates for clusters with strongest negative correlation with log-transformed pain duration.
