# Supplementary material for: Cerebral Cortical Thickness in Chronic Pain Due to Knee Osteoarthritis: The Effect of Pain Duration and Pain Sensitization
Source: PLoS One. 2016 Sep 22;11(9):e0161687. doi: 10.1371/journal.pone.0161687 (PMC5033394; doi:10.1371/journal.pone.0161687)
Supplement: S1 Table — (DOCX) [file pone.0161687.s002.docx]

**Supplementary Table 1:** Characteristics of Knee OA patients

| ID | Sex | Age (Y) | Handedness | Educ. level | Pain duration (Y) | PRI | PPI | Pain-DETECT | BDI | MoCA | Dominant knee | Medications |
| --- | --- | --- | --- | --- | --- | --- | --- | --- | --- | --- | --- | --- |
| 1 | Male | 68 | L | 4 | 10 | 48 | 21 | 25 | 7 | 28 | L | Paracetamol |
| 2 | Female | 72 | R | 3 | 20 | 26 | 19 | 16 | 1 | 27 | R | Atenolol, Simvastatin |
| 3 | Female | 54 | R | 8 | 38 | 8 | 16 | 15 | 5 | 26 | L | Tranexamic acid |
| 4 | Male | 77 | R | 6 | 15 | 29 | 25 | 9 | 9 | 27 | L | Thyroxin, Omeprazole, Silenium |
| 5 | Female | 45 | L | 4 | 4 | 21 | 16 | 15 | 7 | 26 | R | None |
| 6 | Male | 66 | R | 4 | 15 | 8 | 13 | 5 | 14 | 29 | R | None |
| 7 | Female | 54 | R | 4 | 2 | 10 | 14 | 0 | 2 | 25 | R | None |
| 8 | Female | 59 | NA | NA | 25 | NA | NA | 19 | 7 | 20 | L | None |
| 9 | Female | 59 | R | 4 | 3 | 15 | 19 | 12 | 1 | 28 | L | None |
| 10 | Male | 80 | R | 3 | 8 | 6 | 16 | 8 | 7 | 29 | L | None |
| 11 | Female | 71 | R | 6 | 14 | 15 | 15 | 6 | 1 | 26 | L | Paracetamol |
| 12 | Male | 65 | R | 8 | 5 | 12 | 16 | 6 | 1 | 27 | L | None |
| 13 | Female | 70 | R | 6 | 10 | 30 | 20 | 14 | 19 | 29 | L | Losartan, Bendroflumethiazide, Prochlorperazine |
| 14 | Male | 72 | R | 2 | 12 | 0 | 16 | 16 | 4 | 24 | L | Atropine eye drops |
| 15 | Female | 63 | R | 3 | 20 | 22 | 19 | 12 | 13 | 29 | R | Paracetamol |
| 16 | Male | 56 | R | 3 | 10 | 13 | 15 | 12 | 5 | 30 | R | None |
| 17 | Male | 67 | R | 4 | 10 | 10 | 12 | 7 | 7 | 28 | R | Glucosamine, Bendroflumethiazide, Simvastatin |
| 18 | Male | 80 | R | 2 | 10 | 16 | 12 | 17 | 8 | 27 | L | Amlodipine |
| 19 | Male | 57 | R | 8 | 10 | 10 | 16 | 10 | 8 | 29 | R | None |
| 20 | Male | 63 | R | 6 | 9 | 0 | 21 | 1 | 0 | 29 | R | Paracetamol, Aspirin, Co-codamol, Lansoprazole, Glucosamine |
| 21 | Female | 64 | R | 6 | 3 | 24 | 19 | 23 | 0 | 29 | R | Enalapril, Levothyroxine, Bendroflumethiazide, Amitriptyline |
| 22 | Male | 61 | R | 3 | 1 | 8 | 14 | 6 | 2 | 29 | R | None |
| 23 | Male | 63 | R | 2 | 5 | 5 | 14 | 6 | 1 | 30 | L | None |
| 24 | Female | 65 | R | 1 | 6 | 6 | 24 | 10 | 10 | 22 | L | None |
| 25 | Male | 65 | R | 6 | 3 | 26 | 12 | 15 | 4 | . | R | None |
| 26 | Female | 67 | R | 1 | 5 | 10 | 17 | 4 | 5 | 30 | R | None |
| 27 | Male | 48 | R | 3 | 7 | 7 | 16 | 12 | 13 | 27 | R | None |
| 28 | Female | 65 | R | 3 | 7.5 | 25 | 17 | 18 | 13 | 27 | R | Amlodipine |
| 29 | Female | 72 | R | 2 | 5 | 21 | 20 | 18 | 14 | 28 | R | Ramipril, Atorvastatin |
| 30 | Male | 65 | R | 3 | 2.5 | 56 | 20 | 9 | 8 | 26 | R | Amlodipine |
| 31 | Female | 56 | R | 7 | 1 | 21 | 21 | 10 | 0 | 30 | L | None |
| 32 | Female | 83 | R | 3 | 2.5 | 36 | 16 | 2 | 4 | NA | R | None |
| 33 | Male | 71 | R | 2 | 4 | 15 | 15 | 6 | 6 | 26 | R | Paracetamol, Felodipine, Ramipril, Atenolol, Furosemide |
| 34 | Female | 72 | R | 1 | 2 | 20 | NA | 13 | 0 | 18 | L | Oxybutylin |
| 35 | Male | 77 | R | 1 | 20 | 21 | 25 | 12 | 10 | 23 | R | None |
| 36 | Female | 65 | R | 2 | 3 | 2 | 17 | 23 | 19 | 21 | L | None |
| 37 | Female | 67 | R | 2 | 3 | 23 | 21 | 3 | 12 | 30 | R | None |
| 38 | Female | 61 | R | 8 | 1 | 46 | 22 | 15 | 22 | 27 | L | Thyroxin, certaline |
| 39 | Female | 78 | R | 3 | 1 | 28 | 19 | 7 | 12 | 27 | L | None |
| 40 | Male | 67 | R | 3 | 1 | 17 | 18 | 19 | 13 | 28 | L | Paracetamol |

Handedness= based on Edinburgh Handedness Inventory*, Educational level= 1-8 (1 is post-graduate levels and 8 is none*, PRI and PPI= Pain Rating and Present Pain Indices from McGill pain questionnaire*, PainDETECT*, BDI= Beck’s Depression Inventory*. MoCA= Montreal Cognitive Assessment*.

******References are provided accordingly in the main text.***
