# Supplementary material for: Cerebral Cortical Thickness in Chronic Pain Due to Knee Osteoarthritis: The Effect of Pain Duration and Pain Sensitization
Source: PLoS One. 2016 Sep 22;11(9):e0161687. doi: 10.1371/journal.pone.0161687 (PMC5033394; doi:10.1371/journal.pone.0161687)
Supplement: S2 Table — (DOCX) [file pone.0161687.s003.docx]

**Supplementary Table 2:** Detailed description of regions that exhibited significant negative correlation with pain duration in knee OA patients

| **Cluster** | **Sub-clusters** |
| --- | --- |
| **R** | **Posterior ramus of lateral sulcus, temporal plane, superior temporal gyrus, supramarginal gyrus, post-central sulcus, intraparietal sulcus, angular gyrus, subcentral gyrus, superior parietal lobule, parieto-occipital sulcus, cuneus, calcarine sulcus, precuneus, superior occipital gyrus, superior parietal lobule** |
|  | **Fronto-marginal gyrus and sulcus, transverse fronto-polar gyri and sulci, orbital gyri and sulci, inferior and middle frontal sulci, inferior part of the precentral sulcus, precentral gyrus, middle frontal gyrus, superior frontal gyrus and sulcus, anterior part of the cingulate gyrus and sulcus, middle-anterior and middle posterior parts of the cingulate gyrus and sulcus** |
|  | **Inferior frontal sulcus, inferior part of the precentral, sulcus, opercular part of the inferior frontal gyrus** |
|  | **Temporal pole** |
|  | **Marginal branch of the cingulate sulcus, precuneus** |
|  | **Anterior transverse temporal gyrus (of Heschl), inferior segment of the circular sulcus of the insula** |
|  | **Superior temporal sulcus (parallel sulcus)** |
|  | **Middle temporal gyrus** |
|  | **Opercular part of the inferior frontal gyrus** |
| **L** | **Lateral aspect of the superior temporal gyrus, superior temporal sulcus, temporal plane, posterior ramus of the lateral sulcus, supramarginal gyrus, post-central sulcus, superior parietal lobule, intraparietal sulcus, angular gyrus, sulcus intermedius primus, parieto-occipital sulcus, precuneus, subparietal sulcus, posterior-dorsal and middle-posterior parts of the cingulate gyrus and sulcus, marginal branch of the cingulate sulcus, posterior-ventral part of the cingulate gyrus and para-central lobule and sulcus** |
|  | **Posterior medial part of superior frontal gyrus** |
|  | **Superior part of inferior frontal sulcus** |
|  | **Superior anterior part of frontal gyrus** |
